# Supplementary material for: Mapping the evolution of fertility support policies in China: A content and instrumental analysis
Source: PLoS One. 2025 Oct 9;20(10):e0332137. doi: 10.1371/journal.pone.0332137 (PMC12510515; doi:10.1371/journal.pone.0332137)
Supplement: S1 Appendix — (ZIP) [file pone.0332137.s001.zip › S1 Appendix. 226 original policy documents/8-国家人口计生委关于印发《全国流动人口计划生育服务管理工作规范》的通知(FBM-CLI-4-135639).doc]

国家人口计生委关于印发《全国流动人口计划生育服务管理工作规范》的通知

**发布部门：** [国家人口和计划生育委员会(含国家计划生育委员会)(已撤销)](https://www.pkulaw.com/law/chl?Aggs.IssueDepartment=60304) [机构沿革](javascript:void(0))

**发文字号：**国人口发〔2009〕49号

**发布日期：**2009.06.12

**实施日期：**2009.06.12

**时效性：** [现行有效](https://www.pkulaw.com/law/chl?Aggs.TimelinessDic=01)

**效力级别：** [部门工作文件](https://www.pkulaw.com/law/chl?Aggs.EffectivenessDic=XE0303)

**法规类别：** [人口计划管理](https://www.pkulaw.com/law/chl?Aggs.Category=09701)

国家人口计生委关于印发《全国流动人口计划生育服务管理工作规范》的通知
（国人口发〔2009〕49号）

各省、自治区、直辖市人口计生委，计划单列市、新疆生产建设兵团人口计生委，解放军、武警部队人口和计划生育领导小组办公室，中直机关、中央国家机关人口计生委，委机关各单位，各直属、联系单位：
　　《全国流动人口计划生育服务管理工作规范》已经2009年6月10日国家人口计生委第26次主任会议讨论通过，现印发给你们，请认真做好学习培训、贯彻执行和监督检查工作。

　　二〇〇九年六月十二日

　　全国流动人口计划生育服务管理工作规范

　　为提高流动人口计划生育服务管理规范化、制度化水平，切实维护流动人口合法权益，依据《[流动人口计划生育工作条例](https://www.pkulaw.com/chl/f6b2c35d55b7a40ebdfb.html)》，特制定本规范。
　　**一、**基本要求
　　（一）树立以人为本理念，坚持服务管理并重、寓管理于服务之中，实行流动人口与户籍人口同等对待，做到同宣传、同服务、同管理。
　　（二）营造宣传教育氛围，深入宣传计划生育方针政策，广泛普及避孕节育、优生优育、生殖健康科普知识，引导流动人口树立新型婚育观。
　　（三）坚持分类指导，推动体制机制创新，建立和完善流动人口计划生育"统筹管理、服务均等、信息共享、区域协作、双向考核"新机制和"统一管理、优质服务"新体制。
　　（四）坚持依法行政，建立健全流动人口计划生育法规、规章和工作制度，落实户籍地和现居住地责任，促进服务质量和工作水平的提高。
　　（五）加强流动人口信息化建设，做好PADIS流动人口计划生育信息管理系统的建设和应用，实施流动人口信息化服务管理。

　　**二、**工作机制
　　（一）统筹管理。
　　1．建立健全各级流动人口计划生育工作领导保障机制，确保各级流动人口计划生育工作机构设置、人员配备、经费投入能够满足工作要求。
　　2．协调与公安、民政、人力资源社会保障、住房城乡建设、卫生、工商、统计、教育、财政等相关部门的流动人口服务管理，形成人口计生部门与相关部门共同做好流动人口计划生育工作的合力。
　　3．加强人口计生部门内部及人口计生系统工作的统筹协调，坚持属地化、规范化和信息化管理，形成本部门、本系统"一盘棋"的良好工作局面。
　　4．立足社区，健全服务网络，创新服务方式，规范服务行为，依法提供服务，强化服务功能，夯实流动人口计划生育基层基础。
　　5．充分发挥计划生育协会桥梁纽带作用，完善基层组织网络，开展经常性活动，引导流动人口自觉实行计划生育。
　　（二）服务均等。
　　1．流动人口享有现居住地的计划生育服务设施、文化产品等服务资源，逐步实现流动人口计划生育、优生优育和生殖健康基本公共服务均等化。
　　2．按照便民维权的原则，简化办事程序，提高办事效率，落实便民服务措施，切实保障流动人口实行计划生育的合法权益。
　　3．探索建立促进流动人口基本公共服务均等化保障机制，实现流动人口免费计划生育技术服务基本覆盖。
　　（三）信息共享。
　　1．加强流动人口信息采集工作，逐步建立和完善全员流动人口计划生育信息管理系统。
　　2．推进户籍地和现居住地流动人口计划生育信息实时沟通，实现动态服务管理。
　　3．加强人口计生部门与相关部门的协调配合，逐步实现流动人口信息共享。
　　（四）区域协作。
　　1．加强流动人口户籍地和现居住地的协作配合，实行流动人口计划生育双向服务管理。
　　2．流动人口相对聚集的地区，通过签订区域协作协议和召开联席会议等形式，积极开展联合执法和综合服务等活动，建立协作双方或多方相互协调和齐抓共管的流动人口计划生育区域协作机制。
　　3．协作双方或多方加强信息通报、个案处理、政策告知和层级交流等工作，及时相互通报流动人口计划生育工作相关信息；提供发现的涉及"两非"线索，沟通相关信息；协作处理生育服务登记、社会抚养费征收等事宜；通过官方网站、电子或纸质文件等方式告知新出台的政策措施；相应建立省、市、县级交流制度，研究解决实际问题。
　　（五）双向考核。
　　1．将流动人口计划生育纳入人口和计划生育目标管理责任制，开展年度考核，落实"一票否决"。
　　2．对流动人口计划生育工作，实行流入、流出地双向考核、责任共担，从流出地掌握的情况，考核流入地的职责落实情况；从流入地掌握的情况，考核流出地的职责落实情况。
　　3．考核指标以《国家人口计生委关于印发流动人口计划生育管理和服务工作检查评估指标（试行）的通知》（国人口发〔2007〕115号）为主要依据。

　　**三、**工作职责
　　（一）县级以上人口计生部门主要职责。
　　1．宣传贯彻流动人口计划生育法律法规和方针政策，制定本地区流动人口计划生育工作规划和相关制度。
　　2．将流动人口计划生育服务管理纳入本地区人口和计划生育目标管理责任制，组织实施双向考核评估。
　　3．强化流动人口计划生育信息管理系统的建设和应用，做好信息交流、数据分析和统计上报等工作。
　　4．开展区域间流动人口计划生育服务管理协作，协调解决重点难点问题及相关事宜。
　　5．深入基层调查研究，加强业务指导和人员培训，组织开展服务管理专项活动。
　　6．协调相关部门齐抓共管，共同做好流动人口计划生育综合服务管理工作。
　　7．受理并及时处理与流动人口计划生育工作有关的举报，保护流动人口相关权益。
　　（二）乡（镇、街道）主要职责。
　　1．落实流动人口计划生育法律法规和规章制度。
　　2．开展经常性宣传教育活动，提供政策法规、计划生育和生殖健康咨询等服务。
　　3．做好全员流动人口信息的汇总、录入和上报工作，及时通过流动人口计划生育信息管理系统进行通报和反馈。
　　4．及时办理和查验婚育证明，做好流动人口生育登记工作。
　　5．组织从事计划生育技术服务的机构指导流动育龄夫妻选择安全、有效、适宜的避孕节育措施，依法为流动育龄夫妻免费提供国家规定的基本项目的计划生育技术服务。
　　6．依法落实流动人口计划生育奖励和优惠政策，帮助流动人口解决生产、生活、生育等方面的实际困难。
　　（三）村（居）工作要求。
　　1．将流动人口计划生育宣传教育工作融入社区文化建设，普及相关政策法规、生殖健康和避孕节育等知识，落实各项制度和措施。
　　2．负责全员流动人口信息的采集、核实和登记，建立健全计划生育档案，全面和及时掌握相关信息。
　　3．协助乡（镇、街道）做好流动人口计划生育技术服务，动员和指导流动已婚育龄夫妻落实避孕节育措施，组织参加孕情、环情检查，做好药具发放和随访服务工作。
　　4．协助乡（镇、街道）办理和查验婚育证明，指导已婚育龄夫妻办理生育服务登记有关事宜及出具相关证明。
　　5．依托自治网络，整合社区资源，为流动人口送政策、送知识和送服务上门，开展"关怀关爱"等便民利民系列活动。

　　**四、**户籍地服务管理
　　（一）登记建档。
　　1．村（居）对流出人口实行全员登记并采集相关信息，上报乡（镇、街道）录入流动人口计划生育信息管理系统。
　　2．乡（镇、街道）及村（居）定期对辖区内流出人口进行巡查和跟踪服务，及时掌握变化情况和对信息进行更新。
　　3．村（居）建立流出人口信息登记簿、计划生育信息采集卡和其他情况记载资料等档案。
　　4．乡（镇、街道）建立婚育证明登记表、现居住地反馈的《流动人口避孕节育情况报告单》和统计报表等档案。
　　（二）婚育证明办理。
　　1．乡（镇、街道）及时免费为流出育龄妇女办理婚育证明。
　　2．对已流出但未办理婚育证明的育龄妇女，经核实婚育情况清楚的，可由家人为其代办。
　　3．婚育证明有效期为三年，不得以任何理由缩短期限。有效期满的，凭原婚育证明和本人居民身份证免费换领新证。已落实绝育措施且婚育情况未发生变化的，婚育证明长期有效。
　　（三）技术服务。
　　1．在已婚育龄妇女外出前，指导其选择安全、有效、适宜的避孕节育措施。
　　2．告知流出已婚育龄妇女按时参加现居住地的孕情、环情检查。
　　3．对流出已婚育龄妇女接受计划生育技术服务的，在其婚育证明上作相应记录并加盖公章。
　　（四）奖励优惠。
　　依法落实流出人口应享有的计划生育奖励和优惠政策，主要包括：为独生子女家庭办理独生子女父母光荣证并落实相关奖励；为符合农村计划生育家庭奖励扶助、"少生快富"工程和计划生育家庭特别扶助条件的家庭落实奖励政策；协助相关部门为计划生育家庭提供就业、就医和子女入托入学等方面的优惠。

　　**五、**现居住地服务管理
　　（一）登记建档。
　　1．村（居）对流入人口实行全员登记并采集相关信息，上报乡（镇、街道）录入流动人口计划生育信息管理系统。
　　2．乡（镇、街道）及村（居）定期对辖区内流入人口进行巡查和随访服务，及时掌握变化情况和对信息进行更新。
　　3．村（居）建立流入人口信息登记簿、计划生育信息采集卡和其他情况记载资料等档案。
　　4．乡（镇、街道）建立流入已婚育龄妇女孕情、环情检查登记簿、《流动人口避孕节育情况报告单》存根、一孩生育服务登记表和统计报表等档案。
　　（二）婚育证明查验。
　　1．村（居）协助乡（镇、街道）及时查验辖区内流入育龄妇女的婚育证明。
　　2．流入育龄妇女的婚育或避孕节育情况发生变化的，应在婚育证明上作相应记录并加盖公章。
　　3．婚育证明首次查验后，每年至少查验一次。
　　（三）技术服务。
　　1．实行计划生育的流动育龄夫妻免费享有的技术服务项目包括：发放避孕药具；孕情、环情检查；放置、取出宫内节育器及技术常规所规定的各项医学检查；人工终止妊娠术及技术常规所规定的各项医学检查；输卵管结扎术、输精管结扎术及技术常规所规定的各项医学检查；计划生育手术并发症诊治等。
　　2．在流动人口相对聚集的地区设置避孕药具发放点，有条件的在适当位置设置避孕套自动售货机，方便流动育龄夫妻获取避孕药具。
　　3．乡（镇、街道）组织当地从事计划生育技术服务的机构（包括计划生育技术服务机构和从事计划生育技术服务的医疗保健机构，应具备法定资质和条件），为流入已婚育龄妇女开展孕情、环情检查，按规定出具《流动人口避孕节育情况报告单》。
　　4．乡（镇、街道）主动与流动人口户籍地乡（镇、街道）建立联系，依据流入已婚育龄妇女避孕节育情况证明，及时向其户籍地乡（镇、街道）通报流动已婚育龄妇女避孕节育情况。
　　（四）权益保障。
　　为流动人口提供与户籍人口同等的计划生育、优生优育和生殖健康服务，采取多种措施协助解决流动人口在就业、就医、子女入托入学、法律援助和贫困救助等方面的实际问题，维护流动人口合法权益。

　　**六、**协作服务管理
　　（一）信息管理。
　　1．建设和完善国家和省级流动人口计划生育信息管理系统。国家负责跨省流动人口信息的管理工作；省级负责省内流动人口信息的管理工作。
　　2．国家和省级人口计生部门对流动人口计划生育信息管理系统运行情况建立定期通报制度，加强信息动态监测和信息综合分析，促进基层信息管理和应用水平的提高。
　　3．各级人口计生部门配备流动人口服务管理所需计算机及配套设备，加强工作人员的信息管理业务培训，及时准确做好流动人口计划生育信息的录入、更新、通报、反馈和报表上传等工作。
　　4．县、乡信息管理人员及时登录流动人口计划生育信息管理系统，处理流动人口计划生育信息。
　　5．实行流动人口服务管理情况逐级统计上报制度，做好流动人口统计工作。
　　6．各省（区、市）在本级人口计生委网站上，公布本行政区域范围内具有计划生育技术服务资格的技术服务机构名称、地址和联系电话等信息。
　　（二）生育服务。
　　1．对拟在现居住地办理一孩生育服务登记的流动育龄夫妻，凭夫妻双方的居民身份证、结婚证和户籍地乡（镇、街道）出具的婚育情况证明，原则上由女方现居住地按照当地有关规定予以办理。
　　（1）信息核实：
　　①现居住地乡（镇、街道）收到流动育龄夫妻拟生育第一个子女的相关材料后，在7个工作日内通过电话、信函或流动人口计划生育信息管理系统等形式，向夫妻双方户籍地乡（镇、街道）核实其婚育情况。
　　②户籍地乡（镇、街道）接到核实要求后，在15个工作日内予以反馈。
　　（2）服务登记：
　　①现居住地乡（镇、街道）对证明材料齐全的，即时办理一孩生育服务登记。
　　②证明材料不全或有误的，暂缓办理；不符合生育政策的，不予办理，并向当事人书面说明理由。
　　（3）情况通报：
　　①现居住地乡（镇、街道）自办理一孩生育服务登记之日起，在15个工作日内向流动育龄夫妻户籍地乡（镇、街道）予以通报。
　　②由户籍地乡（镇、街道）办理的一孩生育服务登记，自办理之日起，在15个工作日内向流动已婚育龄妇女现居住地乡（镇、街道）予以通报。
　　2．流动人口申请再生育的，由女方户籍地负责审批。生育政策和审批程序依据受理户籍地的有关规定执行。受理申请的户籍地将审批结果在15个工作日内通报其现居住地。
　　（三）协商征收社会抚养费。
　　1．现居住地或发现地县级人口计生部门或其依法委托的乡（镇、街道）在作出征收决定之前，必须由县级人口计生部门先行与政策外生育当事人户籍地县级人口计生部门进行协商。
　　2．协商时间：户籍地自收到现居住地或发现地的相关文本和信息之日起7个工作日内作出回复。
　　3．协商内容：
　　（1）核实当事人的基本情况；
　　（2）了解当事人的实际收入；
　　（3）通报本地城镇居民年人均可支配收入和农村居民年人均纯收入情况；
　　（4）拟征收社会抚养费的方案；
　　（5）需要协商的其他事项。
　　4．协商凭据：协商的双方可以采用公函、传真、电话等方式并提供具有证据效力的原始记录。
　　5．协商时间的计算：公函以挂号签收日期为到达时间；传真以注明接收时间为到达时间；电话以原始记录的通话时间为到达时间。
　　（四）协作监管。
　　1．户籍地与现居住地建立健全同级协商、上级协调、相互配合的协作机制，共同做好流动人口计划生育工作。
　　2．省、市人口计生部门负责本行政区域内流动人口计划生育服务管理的协调和监管工作。
　　3．国家人口计生委负责省际间流动人口计划生育服务管理的协调和监管工作。

　　**七、**工作保障
　　（一）地方各级政府按照与户籍人口人均计划生育经费同等投入水平，将流动人口计划生育服务管理经费纳入财政预算予以保障。
　　（二）省、市、县人口计生部门根据本地流动人口规模设置流动人口计划生育服务管理机构；乡（镇、街道）根据流动人口计划生育工作需要配备专（兼）职工作人员；村（居）按辖区流动人口数量配备所需的流动人口计划生育协管员。
　　（三）各级人口计生部门实行流动人口计划生育工作"一把手"亲自抓、负总责，明确部门内各业务机构及直属单位在做好流动人口计划生育工作中的职责，落实各项工作任务。
　　（四）本规范的执行与落实情况，纳入各级人口和计划生育目标管理责任制，作为平时经常性检查和年终考核评估的重要依据。国家人口计生委定期对各省（区、市）流动人口计划生育服务管理工作进行检查和评估。
　　附件：流动人口计划生育服务管理工作流程图（略）

© 北大法宝：（[www.pkulaw.com](https://www.pkulaw.com/)) 专业提供法律信息、法学知识和法律软件领域各类解决方案。北大法宝为您提供丰富的参考资料，正式引用法规条文时请与标准文本核对。
欢迎查看所有[产品和服务](https://www.pkulaw.com/helps/2.html)。法宝快讯：[如何快速找到您需要的检索结果？](https://www.pkulaw.com/helps/70.html)[法宝](https://www.pkulaw.com/helps/69.html)V6有何新特色？

扫描二维码阅读原文

**原文链接：**<https://www.pkulaw.com/chl/f789654bfbcb19a0bdfb.html>
